# Physiological and Transcriptomic Analyses Reveal Commonalities and Specificities in Wheat in Response to Aluminum and Manganese

**DOI:** 10.3390/cimb46010024

**Published:** 2024-01-02

**Authors:** Daozhen Luo, Chunnuan Xian, Wenjie Zhang, Ying Qin, Qing Li, Muhammad Usman, Shiheng Sun, Yongxiu Xing, Dengfeng Dong

**Affiliations:** Guangxi Key Laboratory of Agro-Environment and Agric-Products Safety, College of Agriculture, Guangxi University, Nanning 530004, China; 2117303008@st.gxu.edu.cn (D.L.); 1917303009@st.gxu.edu.cn (C.X.); 2117401013@st.gxu.edu.cn (W.Z.); yingqinemail@163.com (Y.Q.); 2217303006@st.gxu.edu.cn (Q.L.); usmankhan6191@gmail.com (M.U.); hhsunshiheng@163.com (S.S.); xyx@gxu.edu.cn (Y.X.)

**Keywords:** cell wall, nicotianamine, transcription factors, WGCNA

## Abstract

Aluminum (Al) and manganese (Mn) toxicity are the top two constraints of crop production in acid soil. Crops have evolved common and specific mechanisms to tolerate the two stresses. In the present study, the responses (toxicity and tolerance) of near-isogenic wheat lines (ET8 and ES8) and their parents (Carazinho and Egret) to Al and Mn were compared by determining the physiological parameters and conducting transcriptome profiling of the roots. The results showed the following: (1) Carazinho and ET8 exhibited dual tolerance to Al and Mn compared to Egret and ES8, indicated by higher relative root elongation and SPAD. (2) After entering the roots, Al was mainly distributed in the roots and fixed in the cell wall, while Mn was mainly distributed in the cell sap and then transported to the leaves. Both Al and Mn stresses decreased the contents of Ca, Mg, and Zn; Mn stress also inhibited the accumulation of Fe, while Al showed an opposite effect. (3) A transcriptomic analysis identified 5581 differentially expressed genes (DEGs) under Al stress and 4165 DEGs under Mn stress. Among these, 2774 DEGs were regulated by both Al and Mn stresses, while 2280 and 1957 DEGs were exclusively regulated by Al stress and Mn stress, respectively. GO and KEGG analyses indicated that cell wall metabolism responds exclusively to Al, while nicotianamine synthesis exclusively responds to Mn. Pathways such as signaling, phenylpropanoid metabolism, and metal ion transport showed commonality and specificity to Al and Mn. Transcription factors (TFs), such as MYB, WRKY, and AP2 families, were also regulated by Al and Mn, and a weighted gene co-expression network analysis (WGCNA) identified *PODP7*, *VATB2*, and *ABCC3* as the hub genes for Al tolerance and *NAS* for Mn tolerance. The identified genes and pathways can be used as targets for pyramiding genes and breeding multi-tolerant varieties.

## 1. Introduction

Acid soils are found throughout the world. It is estimated that approximately 30~40% of the world’s total land area and more than 50% of the world’s potential arable land have a pH below 5.5; moreover, soil acidification is increasing worldwide [1]. The primary limitations of acid soils are toxic levels of aluminum (Al) and manganese (Mn), as well as suboptimal levels of phosphorous (P) [2].

Aluminum (Al) makes up 7% of the Earth’s crust and is the most abundant metal element. It is fixed in minerals or bound to mineral surfaces in non-acid soils, and thus benign to plant growth. When soil acidifies and the pH drops below 5, Al^3+^ is solubilized into the soil solution. The activity of Al^3+^ in the soil solution increases with the decreasing pH and peaks at pH 4.1 [3]. The primary symptom of Al toxicity is a rapid (beginning within minutes) inhibition of root growth, resulting in a reduced and damaged root system and limited water and mineral nutrient uptake [2].

Manganese is an essential micronutrient and a toxic element for higher plants, with a narrow concentration window between deficiency and toxicity. With a decreasing pH, the amount of exchangeable manganese, mainly in Mn^2+^ form, increases in the soil solution. Mn^2+^ can be readily transported into the root cells and translocated to the shoots, where it is finally accumulated [4]. Mn toxicity symptoms are localized to the shoot, characterized by stunted growth, chlorosis, and necrotic lesions in the leaves, accompanied by the inhibition of photosynthesis and the accumulation of excessive reactive oxygen species (ROS) [5,6].

As the co-existing metal becomes stressed, Al and Mn exert common toxicity effects on the morphology and physiology, such as inhibiting root growth, disturbing ion balance, and overproducing reactive oxygen species, subsequently resulting in oxidative damage, decreasing the chlorophyll content, and deteriorating the photosynthetic performance [2,7,8]. On the other hand, plants have evolved external exclusion and internal tolerance mechanisms to cope with both active Al and excess Mn. External exclusion mechanisms include the chelation of Al and Mn in the rhizosphere by organic acids and second metabolites [9] and the induction of a pH barrier in the rhizosphere [6,10,11]. By contrast, internal tolerance mechanisms enable plants to deal with Al^3+^ and Mn^2+^ once they enter plant cells, either by forming harmless complexes with organic acids, proteins, phenols, and other organic compound ligands, by sequestering them to organelles (e.g., vacuoles, endoplasmic reticula, and the Golgi apparatus), or by rapidly repairing any lesions incurred, including those resulting from oxidative stress [5,6,10].

Due to differences between Al and Mn in the binding site, distribution, and biological functions in plants, some plants’ responses (toxicity and resistance) to Al and Mn are specific [5,9,12] or similar but play different roles and are dominated by various genes. For example, plants transport Al and Mn from the roots to leaves or internally across the cytosol into various organelles, apoplasts, and cell walls through the activities of series transporters, representing a diverse set of metal transporter families [5,13,14]. In addition, the complex gene regulatory network of plant hormones, signal transduction, and transcription factors endows plants with specific and common tolerance mechanisms under Al and Mn stress, such as regulating root growth, cell wall modification, and organic acid secretion [5,15,16].

Traditional strategies used to maintain crop production in acid soils include applying lime to raise the soil pH and planting tolerant crop species and cultivars. Although the liming of soil can ameliorate some Al and Mn toxicity and increase calcium and magnesium concentrations in many acid soils worldwide, it is expensive and ineffective in the subsoil. In some cases, heavy lime application may cause a deleterious effect on the soil structure and reduce the availability of other cationic micronutrients that are essential for plant growth [17,18]. Plant species vary in their tolerance to stresses in acid soils. Native plants usually have more substantial tolerances to adverse factors than crops [19]. Moreover, there is a wide range of tolerance to either Al [20] or Mn [21] among genotypes within the same species. Ideal crop cultivars growing in acid soil should tolerate both Al and Mn toxicity simultaneously. However, plants may employ some specific mechanisms to tolerate Al or Mn despite their coexistence. Many cultivars tolerate only one toxicity [4,21]. Two strategies could be used to obtain duel-tolerant varieties. One approach is to select and breed manganese-tolerant varieties from aluminum-tolerant germplasms, or vice versa, but this is generally restricted by a genetic bottleneck. Another strategy is to design smart plants and pyramid genes to fortify the common ones and compensate for the differential tolerances. Uncovering the commonalities and the specificities of Al and Mn toxicity and tolerant mechanisms is a prerequisite for identifying and pyramiding hub genes and breeding multi-tolerant genotypes. Although the physiological mechanisms by which plants coordinately adapt to multiple stresses in acid soils have been extensively studied [4,21,22], the molecular mechanisms are still largely unknown. Many genes may have co-evolutionary mechanisms in their regulatory pathways and function in the context of multiple stresses.

In the face of increasing environmental challenges, understanding the molecular and physiological responses to abiotic stresses is paramount for ensuring global food security. Aluminum (Al) and manganese (Mn) stresses pose significant threats to crop productivity [4], particularly in wheat, a staple food for a large portion of the world’s population [22]. High-throughput RNA sequencing will be employed to capture the dynamic changes in gene expression profiles, and the transcriptome analysis will provide a genome-wide perspective on the molecular events occurring in wheat plants under Al and Mn stresses [23,24]. This study integrated physiological and transcriptome analyses to reveal the specificity and commonality in the toxicity and tolerance mechanisms of wheat under Al and Mn stresses, providing a new perspective on the molecular network of plants responding to multiple stresses in acid soil and further discovering candidate genes for wheat Al and Mn tolerance, providing genetic resources.

## 2. Materials and Methods

### 2.1. Plant Materials and Treatment

Near-isogenic (over 95%) wheat lines differing in Al tolerance at the *ALMT1* locus, ET8 (homozygous Al-tolerant), and ES8 (homozygous Al-sensitive) [25], as well as their parents Carazinho (Al tolerant) and Egret (Al sensitive), were used as plant materials. The ET8 and ES8 lines were derived from a cross between Carazinho and Egret, with the resulting progeny being backcrossed eight times to Egret or derivatives of Egret. Grain yield increased in Egret-derived lines when Al tolerance from Carazinho was introduced. The seeds were provided by Peter Ryan from CSIRO Plant Industry, Canberra, Australia.

Wheat seeds were surface-sterilized with 0.5% NaClO (*v*/*v*) solution for 20 min, rinsed with sterile water, and allowed to germinate in wet paper towel for 4~5 days at 4 °C before the seedlings were transplanted into continuously aerated Magnavaca hydroponic solution (pH 4.2) for a 2-day acclimation to low pH in a walk-in growth chamber with a stable temperature of 23 °C, a 14 h photoperiod of 400 μmol m^−2^ s^−1^ illumination level, and a relative humidity of 60–80%. Then, the seedlings were stressed by replacing fresh Magnavaca solutions with an addition of 100 µM KAl(SO)_4_ (as Al toxicity), 500 µM MnCl_2_ (as Mn toxicity), or no addition (as control). Free Al^3+^ and Mn^2+^ concentrations in the solution were 25 µM and 267 µM, respectively, as predicted using the WinIAP program (https://www.sequentix.de/index.php#Winiap accessed on 10 October 2023). The experiment was arranged in a completely randomized block design with at least three replicates per batch.

### 2.2. Relative Root Length

Before and four days after stress, the fresh roots of different treatment groups were collected and completely expanded onto the scanner platform (Epson Expression 1000XL, Seiko Epson Corporation, Nagano, Japan). The total length per plant was evaluated using image analysis with WinRhizo Ver. 2.0 (Regent Instruments Inc., Quebec, QC, Canada). The RRL, the ratio of net root growth under Al or Mn treatment compared to the control, was calculated.

### 2.3. SPAD and Brown Spots

After four days of stress, the relative content of chlorophyll in fully developed leaves, as dictated by SPAD (Soil and Plant Analyzer Development) value, was determined with a portable Konica SPAD-502 Plus instrument (Konica Minolta Holdings Inc., Tokyo, Japan). Brown spots on leaves under Mn stress were checked and photographed.

### 2.4. Metal Ions Accumulation and Subcellular Distribution

Al and Mn concentrations in roots and shoots and Ca, Mg, Zn, and Fe concentrations in whole plant were determined after four days of stress. The plant materials were dried in an oven at 105 °C for 1 h, followed by drying at 80 °C for 3 h until a constant weight was achieved. Then, they were ground using a JC-FW100 grinder (Juchuan Co., Qingdao, China). After being dried and pulverized, the plant materials were conventionally digested with mixed acid (concentrated nitric acid/perchloric acid = 4:1) in a microwave digestion instrument (MARS, CEM company, Charlotte, NC, USA) with a digestion procedure of 150 °C for 8 min, 180 °C for 5 min, and 200 °C for 5 min, and then determined using inductively coupled plasma atomic emission spectrometry (ICP-AES, Juguang Co., Beijing, China).

Cell sap in root apices preparation and the extraction of ions (Al and Mn) from the residual cell wall were conducted according to [26]. Al and Mn concentrations were determined by ICP-AES. The mixture of standards was prepared using certified reference materials (CRM) purchased from Sigma-Aldrich (St. Louis, MO, USA) for Al, Mn, Ca, Mg, Zn, and Fe determination.

### 2.5. RNA Extraction, Library Preparation, Sequencing, and Read Mapping

Total RNA was extracted from 1 cm root apices using TRIzol Reagent (Invitrogen Co., Carlsbad, CA, USA) according to the manufacturer’s instructions. The RNA quality was determined using 5300 Bioanalyser (Agilent Co., Santa Clara, CA, USA) and quantified using the ND-2000 (Thermo Fisher NanoDrop, Waltham, MA, USA). Only high-quality RNA sample (OD260/280 = 1.8~2.2, OD260/230 ≥ 2.0, RIN ≥ 6.5, 28S:18S ≥ 1.0, > 1 μg) was used to construct sequencing library and verified by qRT-PCR.

RNA purification, reverse transcription, library construction, and sequencing were performed at Shanghai Majorbio Bio-pharm Biotechnology Co., Ltd. (Shanghai, China) according to the manufacturer’s instructions (Illumina, San Diego, CA, USA). Thirty-six RNA-seq transcriptome libraries (4 varieties × 3 treatments × 3 biological replicates) were prepared and sequenced.

The raw paired-end reads were trimmed and quality controlled using Fastp [27] with default parameters. The clean reads were separately aligned to the wheat genome with orientation mode using HISAT2 software Ver. 2.2.1 [28]. The mapped reads of each sample were assembled using StringTie Ver. 1.3.6 [29] in a reference-based approach. All assembled genes were annotated against Kyoto Encyclopedia of Genes and Genomes (KEGG), Swiss-Prot, Protein family (Pfam), Gene Ontology (GO), Clusters of Orthologous Groups of Proteins (COG), and NCBI non-redundant protein sequences (NR) libraries.

### 2.6. Differential Expression and Functional Enrichment Analysis

The expression level of each transcript was calculated according to the fragments per kilobase of exons model per million mapped reads (FPKM) method. RSEM was used to quantify gene abundances [30]. Differential expression analysis was performed using the DESeq2 [31]. DEGs with |log_2_FC| ≥ 1 and FDR ≤ 0.001 were considered significantly differently expressed genes. Cluster heat diagrams were drawn using Toolkit for Biologists (TB) tools Ver. 2. 030 with default settings [32]. Those data were added to the Comprehensive Gene Expression Database with the accession number PRJNA1031207. In addition, GO annotation and KEGG pathway analysis were carried out using Goatools and KOBAS [33] to identify which DEGs were significantly enriched in GO terms and metabolic pathways at Bonferroni-corrected *p*-value ≤ 0.05 compared with the whole-transcriptome background.

### 2.7. Quantitative Real-Time PCR (qRT-PCR) Validation

Using the identical RNA/cDNAs for RNA-seq as templates, qRT-PCR was performed on a Bio-Rad CFX96 (Bio-Rad Laboratories, Hercules, CA, USA) to verify the authenticity of transcriptomic profile expression patterns. The 10 μL reaction system contained 5 μL of TB Green^®^Premix Ex Taq™ II (Takara standard Co., Osaka, Japan); 10 μM of primers, each 0.2 μL; 1 μL of cDNA template, and 3.6 μL of ddH_2_O. The amplification procedure was initially 95 °C for 30 s, followed by 40 cycles of 95 °C for 10 s, and 60 °C for 30 s (two-step thermal cycling). Twenty-one candidate DEGs involved in various processes were randomly picked up as target genes, and the housekeeping gene *TaActin* was used as an internal control. The primers used for qRT-PCR are listed in Table S1. The relative level of expression was calculated using the 2^−ΔΔCT^ formula [34] (Livak and Schmittgen, 2001).

### 2.8. Weighted Gene Co-Expression Network Analysis (WGCNA)

Co-expression networks were constructed based on pairwise correlations of gene expression across all samples to find clusters (modules) of highly correlated genes and explore the connection of the modules with physiological traits and the hub genes [35]. After filtering out the genes with a low expression (FPKM < 2; CV > 0.15), 9924 genes were retained for the WGCNA. We introduced the appropriate soft threshold power (β = 7) in this study according to scale independence and mean connectivity curves produced using the software. Then, the adjacency was transformed into a topological overlap matrix (TOM), and average linkage hierarchical clustering was conducted according to the TOM-based dissimilarity measure, along with merged genes with a minimum size of 30 and merge cut height of 0.25 as a module. Modules were defined as clusters of highly interconnected genes, and genes within the same cluster had high correlation coefficients among them. The total connectivity and intramodular connectivity were calculated with weighted and correlation coefficients in each module. Biologically meaningful co-expression modules were subsequently analyzed through the heatmap of the correlation coefficients between trait and module. Co-expression network analysis uses the top 30 gene nodes with connectivity, in which each node represents a gene, and the connecting lines (edges) between genes represent co-expression correlations. Interaction network visualization for each module was performed using Cytoscape version 3.9.1 [36]. Hub genes have the highest Kme (Eigengene connectivity) value, indicating the most connections in the network.

### 2.9. Statistical Analysis

Statistical analysis was carried out using SPSS version 19.0 for analysis of variance (ANOVA), followed by Duncan’s multiple range test (DMRT) to compare means among treatments if the ANOVA result was significant (*p* ≤ 0.05). Before ANOVA, the data were tested for normality using Chi-squared analysis and transformed where necessary.

Since *RRL* is a ratio of means, while the measured net root growth values (*x*) were not paired between control and stress groups, the mean value and standard derivation of RRG were calculated according to the following Equation [37]:

$$\bar{RRL}=\frac{{\bar{x}}_{Al\;or\;Mn}}{{\bar{x}}_{CK}};\;{SD}_{RRL}=\bar{RRL}\times \sqrt{{\left(\frac{{SD}_{Al\;or\;Mn}}{{\bar{x}}_{Al\;or\;Mn}}\right)}^{2}+{\left(\frac{{SD}_{CK}}{{\bar{x}}_{CK}}\right)}^{2}}$$

## 3. Results

### 3.1. Tolerance of Wheat Varieties to Al and Mn Toxicity

**Figure 1:**
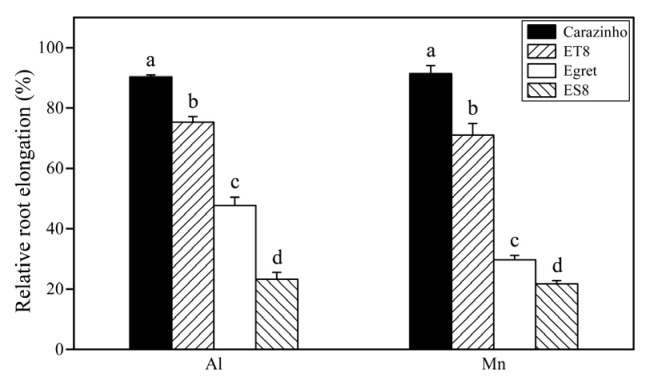
Relative root length of wheat varieties under Al and Mn stresses. Data are expressed as the mean ± standard deviation (SD). Histogram columns within a cluster labeled with different lowercases were significantly different (*p* < 0.05).

The inhibition of root growth, as indicated by the relative root length (RRL), is the initial and primary symptom of early Al toxicity. Aluminum tolerance was precisely the breeding target for creating NILs ES8 and ET8. Not surprisingly, the RRLs of the aluminum-tolerant parent, Carazinho, and the near-isogenic line, ET8, were significantly higher than those of Egret and ES8. Interestingly, the change order of the root length (Carazinho > ET8 > Egret > ES8) under Mn stress was the same as that under Al stress, and the change amplitude was similar (Figure 1).

**Figure 2:**
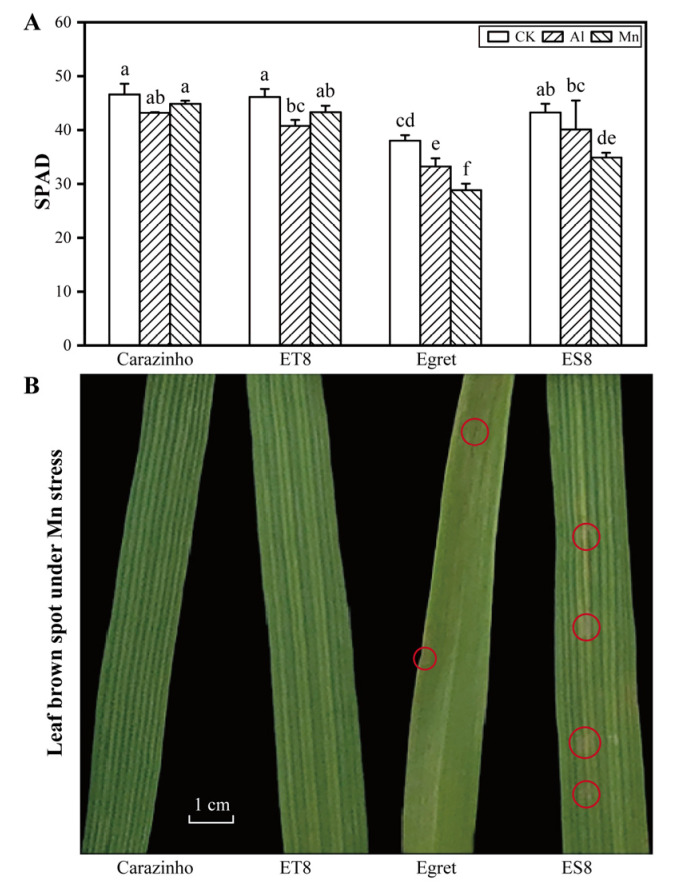
(**A**) Chlorophyll level under Al and Mn stresses. (**B**) Leaf brown spots (labeled in red circles) under Mn stress. Data are expressed as the mean ± standard deviation (SD). Histogram columns within a cluster labeled with different lowercases were significantly different (*p* < 0.05).

Al and Mn toxicity decreased the chlorophyll level, as indicated by the SPAD value, and the decreasing amplitude values in Carazinho and ET8 were lower than those of Egret and ES8. There was no significant difference in the inhibitory effect of Mn and Al in Carazinho and ET8, while Mn showed a more substantial effect than Al in the Egret and ES8 varieties. Furthermore, Mn toxicity symptoms like brown spots on the old leaves or crinkles on the young leaves were found only on the leaves of Egret and ES8 after 4 d of Mn stress (Figure 2).

Collectively, nearly equal damage to the wheat was caused by 25 µM of Al and 267 µM of Mn. The concentrations of active Al and Mn stresses screened from the preliminary experiments were suitable for a further comparison of the phenotypes and mechanisms of the two stresses. The consistency of tolerance to Al and Mn in near-isogenic wheat lines (ET8 and ES8) and their parents (Carazinho and Egret) confirmed the possibility of co-adaptation and breeding multi-resistant varieties.

### 3.2. Al and Mn Subcellular Distribution in Root Cell and Contents in Plants

**Figure 3:**
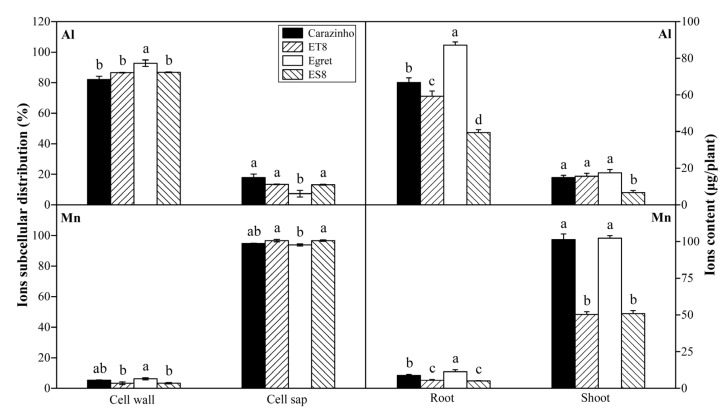
Al and Mn contents and subcellular distribution in root cell. Data are expressed as the mean ± standard deviation (SD). Histogram columns within a cluster labeled with different lowercases were significantly different (*p* < 0.05).

After an influx from the rhizosphere and entry into root cells, Al was mainly fixed in the cell wall, with a proportion variation of 82% to 93% among the genotypes, and only a small part was distributed in the cell saps. The proportions of Al fixed by the cell wall in the tolerant varieties were lower than those of the sensitive varieties, possibly resulting in a low degree of damage to the cell wall rigidification. By contrast, most of the Mn in the root cell was distributed in the cell sap rather than in the cell wall. There was a slight genotypic variation, but there was no correlation to Mn tolerance (Figure 3).

Most of the Al absorbed by the wheat accumulated in the root, and only a small part of Al (<20%) was transported to the shoot, while Mn was transported to the shoot, and only a small amount of Mn (<10%) stayed in the root (Figure 3). The root-to-shoot ratio (R/T) of the Al and Mn contents did not differ significantly among the cultivars. Of note, due to the small biomass, the Mn and Al contents in the root, shoot, and whole plant of ES8 and ET8 were significantly lower than those of their parents, Carazinho and Egret. There was no significant difference in concentration based on dry weight among cultivars.

### 3.3. Al and Mn Effectiveness on Four Metal Ions Contents

**Figure 4:**
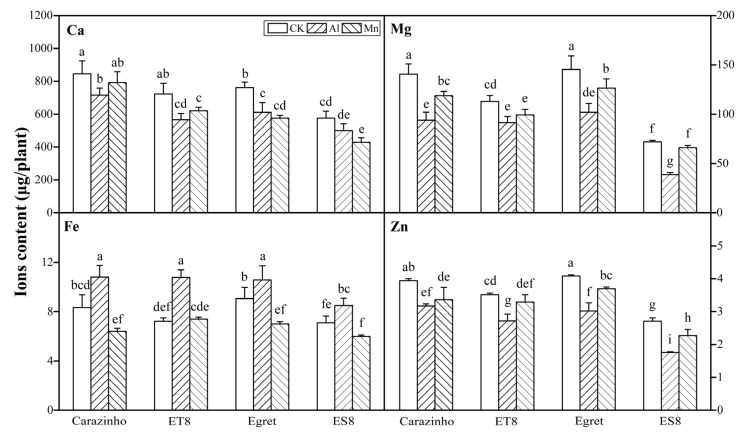
Nutrient element contents in wheat plants under Al and Mn toxicity. Data are expressed as the mean ± standard deviation (SD). Histogram columns labeled with different lowercase letters are significantly different (*p* < 0.05).

The variance analysis results revealed that for every element (Ca, Mg, Fe, and Zn), the contents in wheat were significantly different among stress treatments (F = 27.336~83.02, F_0.01_(2, 24) = 5.61), varieties (F = 10.9~116.62, F_0.01_(3, 24) = 4.72), and their combinations (F = 19.69~52.90, F_0.01_(11, 24) = 3.09). The Ca, Mg, and Zn levels in the wheat were dramatically decreased by both Al and Mn stresses. The decreasing effects of Al and Mn toxicity on the Ca content were roughly equivalent. However, the decreasing effects of Al stress on the Mg and Zn contents were more substantial than those of Mn toxicity. Al toxicity significantly increased the Fe contents, while Mn toxicity had the opposite effect. The significant differences in the Ca, Mg, Fe, and Zn contents among the varieties were not correlated to Al or Mn tolerance. The significance of multiple differences among stress combinations with varieties was labeled in Figure 4.

### 3.4. Wheat Root Transcriptome Profiling in Response to Al and Mn

Whole genome transcriptome sequencing analysis was applied to investigate the molecular responses of wheat roots to Al and Mn toxicity. Thirty-six cDNA libraries were constructed, combining three stresses, four varieties, and three replicates. These libraries yielded 1.8 G clean reads. The Q30 values of all samples exceeded 93.5% with a confidence of 99.9%, and the minimum mapping rate was over 90.55%, indicating that the sequencing was of high quality and qualification for the subsequent gene expression analysis.

A total of 115,534 expressed genes (91,609 known and 23,925 new) and 193,406 transcripts (113,693 known and 79,713 new) were assembled. The functions of 91,313 genes and 113,446 transcripts were finally annotated while referencing the KEGG, Swiss-Prot, Pfam, GO, COG, and NR libraries.

**Figure 5:**
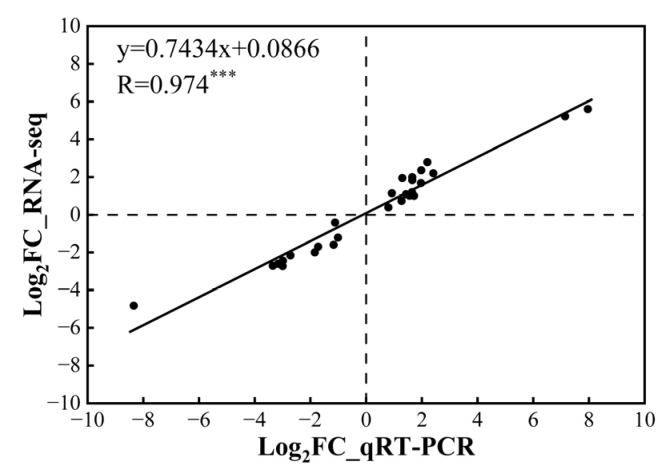
Correlation analysis of differentially expressed genes’ (DEGs) expressions between transcriptome data and qRT-PCR results. *** indicates significance at *p* < 0.001, every black dot represents one gene expressed in the same sample determined by RNA-seq and qRT-PCR methods.

To validate the RNA-seq’s accuracy of gene expression, qRT-PCR was performed using 21 randomly selected genes (Table S1) and identical cDNA templates. The results of the qRT-PCR showed a very significant positive correlation with the transcriptome data, indicating that the transcriptome data were reliable (Figure 5).

### 3.5. Identification of Differentially Expressed Genes in Response to Al and Mn Toxicity

**Figure 6:**
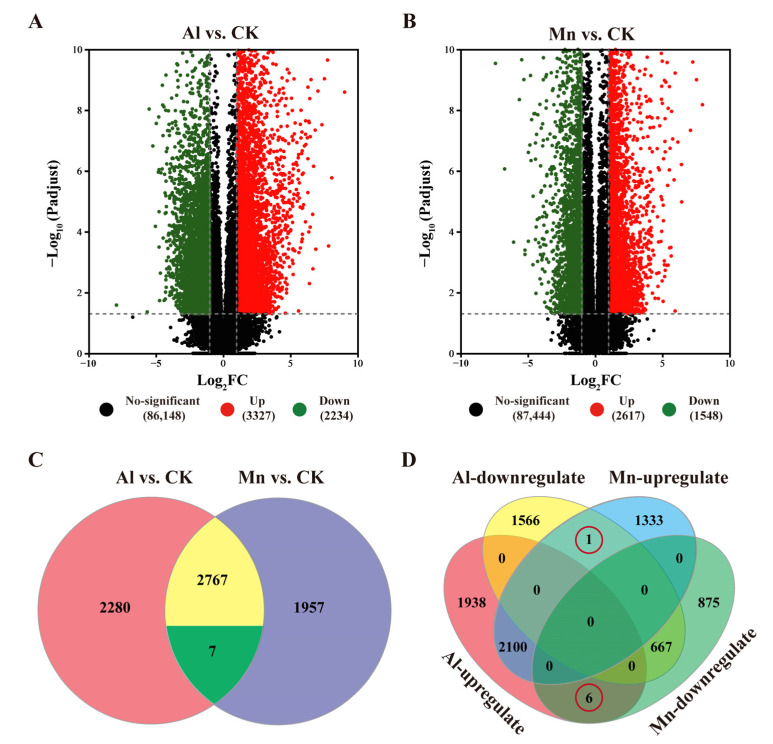
Al and Mn responsive gene expression profiles. The volcanic map of DEGs under Al and Mn stresses, respectively (**A**,**B**). Venn diagrams converge the DEGs under Al and Mn stresses (**C**,**D**).

The FPKMs of genes expressed under Al and Mn toxicity were compared with the control using the criteria |log2FC| ≥ 1 and *p* < 0.001. The DEGs from various varieties were pooled within the stress treatments. A total of 5461 DEGs were identified under Al stress, comprising 3227 upregulated and 2234 downregulated DEGs (Figure 6A). Similarly, 4165 DEGs were identified under Mn stress, comprising 2617 upregulated and 1548 downregulated genes (Figure 6B).

The Venn diagram categorizes the 7699 genes responded by Al and Mn into eight sets, classified into four types based on expression patterns (Figure 6C,D, Table S2). Among these, 1950 genes expressed in the same pattern under Al and Mn toxicity (1283 genes upregulated and 667 genes downregulated by both Al and Mn; Type I) were identified as possible candidate genes contributing to common toxicity or tolerant mechanisms. In contrast, seven genes were differentially expressed under Al and Mn toxicity but in the opposite regulatory direction (Type Il), among which one gene was annotated as a solute carrier family 40 member I, and it was downregulated by Al but upregulated by Mn, and six genes were upregulated by Al but downregulated by Mn, including three genes encoding a bidirectional sugar transporter, one gene encoding ricin B-like lectin, one gene encoding cytochrome P450, and one gene encoding cysteine synthase. In addition, 3504 genes (1938 up, 1566 down) were exclusively regulated by Al (Type Ill), and 1950 genes (1333 up, 875 down) were exclusively regulated by Mn (Type IV). The latter three types of genes were studied to analyze the potential specific toxicity or tolerant mechanisms (Figure 6C,D).

### 3.6. GO Functional Annotations and KEGG Pathway Analysis

**Figure 7:**
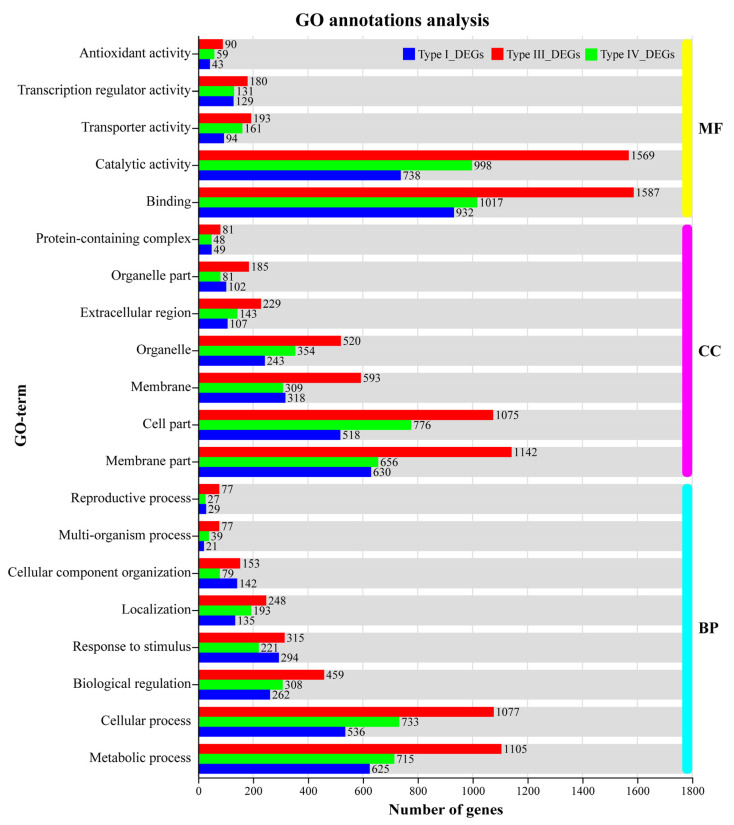
GO annotation analysis of four types of DEGs under Al and Mn stress. Type I_DEGs: genes expressed in the same direction under Al and Mn stresses; Type II_DEGs: 7 genes were expressed in opposite directions but were not annotated; Type III_DEGs: genes exclusively responded to Al; Type IV_DEGs: genes exclusively responded to Mn; MF: molecular function; CC: catalytic activity; BP: biological process.

The GO annotation results showed that binding (GO: 0005488) and catalytic activities (GO: 0003824) in the molecular function (MF) categories; the membrane part (GO: 0044425), cell part (GO: 0044464), and organelle (GO: 0043226) in the cellular component (CC) categories; and the metabolic process (GO: 0008152), cellular process (GO: 0009987), and biological regulation (GO: 0065007) in the biological process (BP) categories were the most abundant DEGs of the GO annotation terms. In the transporter activity (GO: 0005215) term, 193 genes (88 up, 105 down) responded exclusively to Al, 161 genes (95 up, 66 down) exclusively responded to Mn, and 94 genes (49 up, 45 down) responded to both Al and Mn, expressed in different trends. In the transcription regulator activity (GO: 0140110) term, 145 genes (145 up, 35 down) responded exclusively to Al, 161 genes (109 up, 22 down) responded exclusively to Mn, and 94 genes (115 up, 14 down) responded to both Al and Mn, indicating that most transcription factors were upregulated in response to Al and Mn. In the antioxidant activity (GO: 0016209) term, 90 genes (32 up, 58 down) exclusively responded to Al, 59 genes (15 up, 44 down) exclusively responded to Mn, and 94 genes (5 up, 38 down) responded to both Al and Mn (Figure 7).

**Figure 8:**
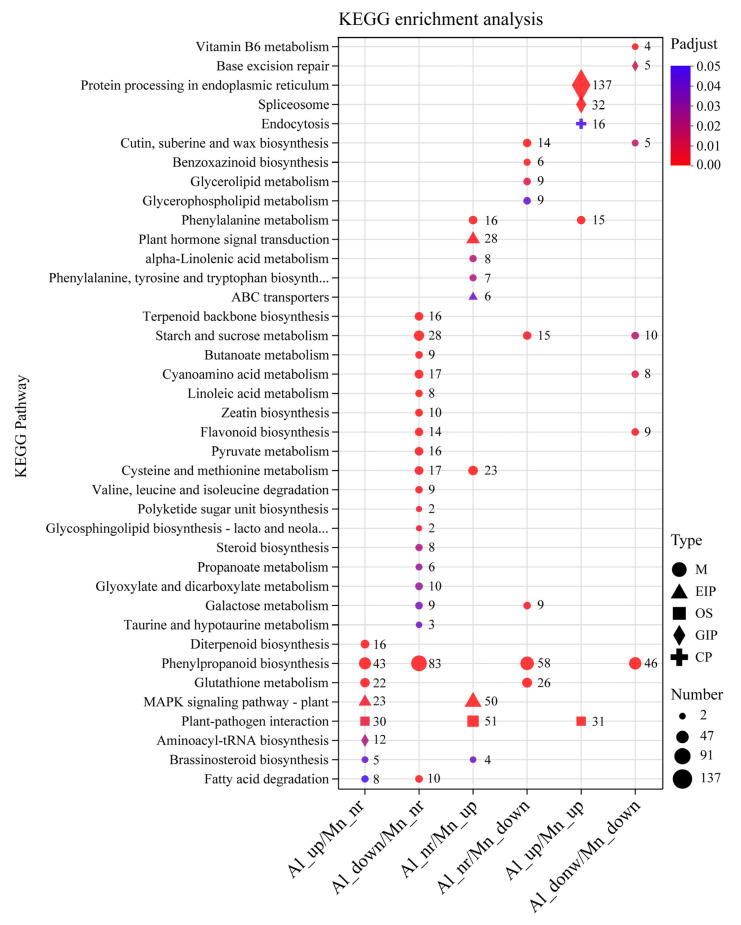
KEGG enrichment for eight DEGs set under Al and Mn stresses. The bubble size indicates the number of genes involved in the terms and pathways. DEGs set under Al_up/Mn_down and Al_down/Mn_up were not enriched into any pathways. Note: Al (Mn)_up: upregulated by Al (Mn); Al (Mn)_down: downregulated by Al (Mn); Al (Mn)_ns: did not respond to Al (Mn); M: metabolism; GIP: genetic information processing; EIP: environmental information processing; OS: organismal systems; CP: cellular process.

The top 20 KEGG enrichment pathways with the smallest FDR values are displayed in Figure 8. The results showed that the genes upregulated by Al or Mn were enriched into five categories, including metabolism (M,14 pathways), genetic information processing (GIP, 4 pathways), environmental information processing (EIP, 2 pathways), organismal systems (OS, 1 pathway), and cellular process (CP, 1 pathway). In contrast, the genes downregulated by Al or Mn were mainly grouped into the metabolism (M) category, which contains 22 pathways. The genes that were upregulated by both Al and Mn were enriched in protein processing in *the* endoplasmic reticulum (GIP, 137 genes), spliceosome (32 GIP), plant–pathogen interaction (OS, 31 genes), endocytosis (CP, 16 genes), and phenylalanine metabolism (M, 15 genes). The genes that were downregulated by both Al and Mn were enriched in seven pathways, such as phenylpropanoid biosynthesis (M, 46 genes), starch and sucrose metabolism (M, 10 genes), and base excision repair (GIPs, 5 genes). Genes that were upregulated by Al were enriched in diterpenoid biosynthesis (M, 16 genes) and aminoacyl-tRNA biosynthesis (GIP, 2 genes). The genes that were downregulated by Al were enriched into 12 pathways, such as fatty acid degradation (M, 10 genes), terpenoid backbone biosynthesis (M, 16 genes), and zeatin biosynthesis (M, 10 genes). Genes that were upregulated by Mn were enriched into plant hormone signal transduction (EIP, 28 genes); alpha-linolenic acid metabolism (M, 8 genes); phenylalanine, tyrosine, and tryptophan metabolism (M, 7 genes); and ABC transporter (6 genes) pathways. Genes that were downregulated by Mn were enriched into cutin, suberine, and wax biosynthesis (M, 14 genes); benzoxazinoid biosynthesis (M, 6 genes); and glycerolipid metabolism (M, 9 genes). Genes that were upregulated by Al and Mn were enriched in the MAPK signaling pathway (EIP, 23 genes upregulated by Al and 50 genes upregulated by Mn) and brassinosteroid biosynthesis (M, 5 genes upregulated by Al and 4 genes upregulated by Mn), and genes that were downregulated by Al and Mn were enriched in galactose metabolism (M, 9 genes downregulated by Al and 9 genes downregulated by Mn). Genes that were enriched in fatty acid (M, 8 genes upregulated by Al and 10 genes downregulated by Mn) and cysteine and methionine metabolism (M, 17 genes downregulated by Al and 23 genes upregulated by Mn) were oppositely regulated by Al and Mn.

### 3.7. Correlations between Physiological Traits and Expressed Module Eigengenes

**Figure 9:**
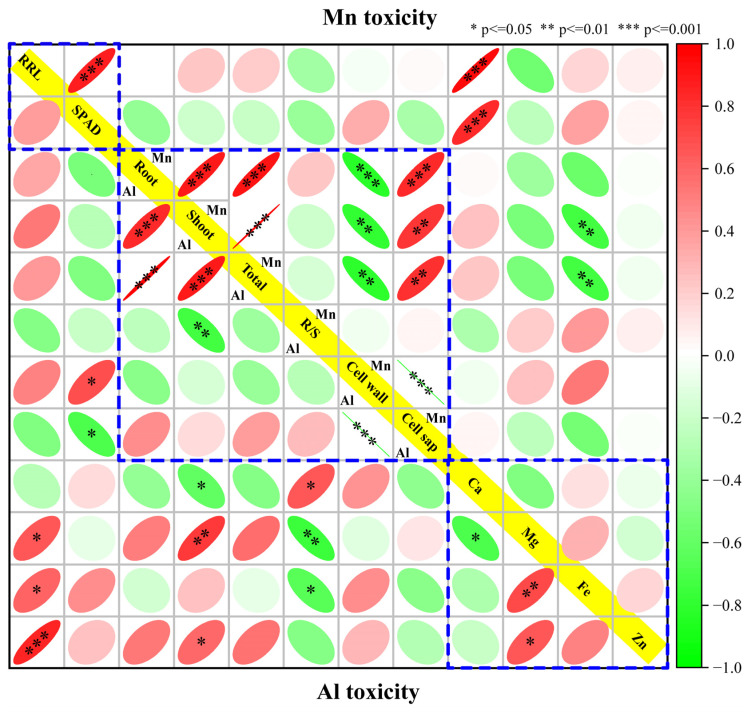
Correlation between tolerance, toxicity, and nutrient ion contents under Al (left bottom) and Mn (right upper) stresses. The parameters in yellow line (from left upper to right bottom) are as follow: relative root length, SPAD, Al or Mn contents in root, shoot, total plant, ratio of root to shoot, cell wall and cell sap, and nutrients (Ca, Mg, Fe, and Zn) content in total plant, respectively.

The correlation analysis showed that RRL and SPAD were significantly positively correlated under Mn stress but not under Al stress. The Al contents in the root, stem, and total plant were significantly positively correlated, while they were not correlated with the distribution ratio between the cell wall and cell sap. Similarly, the Mn contents in the root, stem, and total plant were also significantly positively correlated. Besides that, they all were negatively regulated with the distribution in the cell wall and thus positively correlated with the distribution in the cell sap. There was no significant correlation between the Ca, Mg, Fe, and Zn variation amplitudes under Mn stress. However, the variation of Mg was positively correlated with Ca and negatively correlated with Fe and Zn under Al stress (Figure 9).

**Figure 10:**
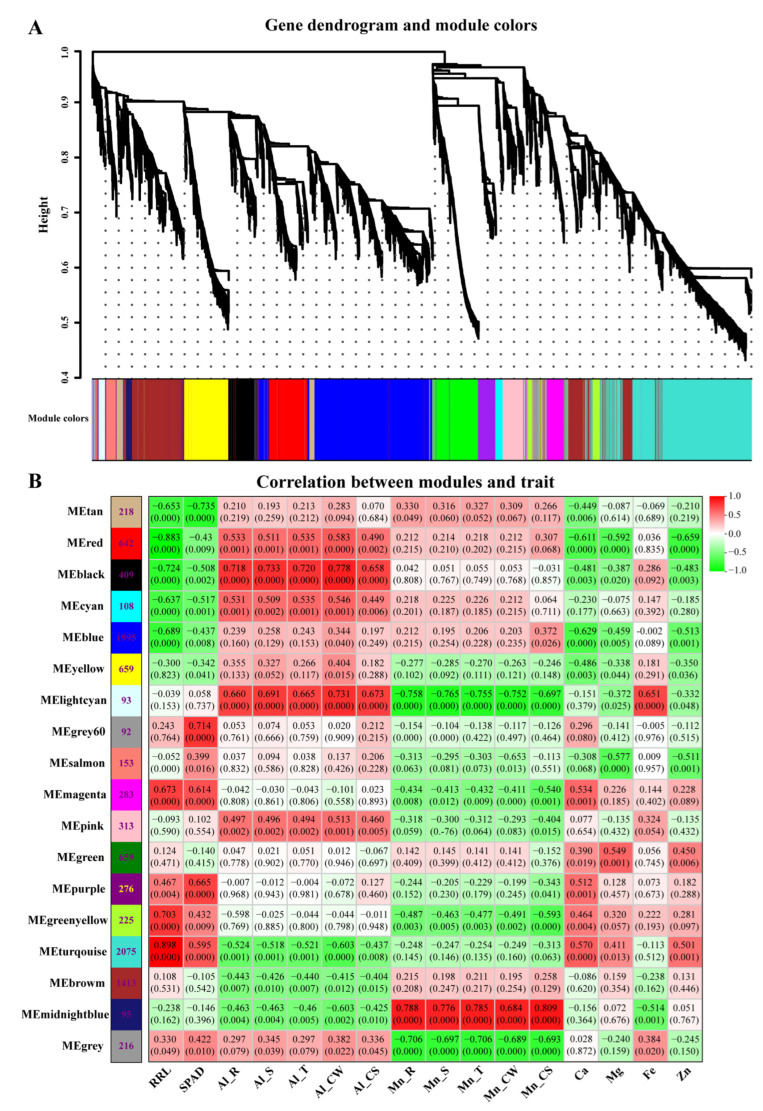
Correlation between gene expression module and physiological trait. (**A**) Hierarchical clustering tree showing co-expression modules. Each leaf in the tree represents one gene. The major tree branches constitute the modules labeled with different colors. (**B**) Matrix of module–trait correlation. A total of 18 modules are shown on the left, and the relevance color scale from −1 to 1 is shown on the right. The high correlation between a specific module and the sample is shown in dark red or dark green. The numbers in parentheses represent the significance (*p*), and the numbers above represent the correlation coefficient (r).

A total of 9924 filtered genes in 36 samples were clustered and divided into 18 module eigengenes (ME) decorated with diacritical colors, respectively, based on a scale-free topological model, β = 7 (Figure 10 and Figure S1). The correlation coefficient and correlation between the module and trait were calculated to explore the genes and expression patterns involved in the physiological trait and tolerance and to find specific expression genes in the samples. Five among eighteen MEs were notably correlated with at least one trait. MEmidnightblue (95 genes) was positively correlated with Mn traits (contents and distribution). MEblack (409 genes) was positively correlated with Al traits (contents and distribution). MElightcyan (93 genes) was positively correlated with Al traits and Fe content, while it was negatively correlated with Mn traits. MEred (642 genes) was negatively correlated with RRL. MEturquoise (2075 genes) was positively correlated with RRL, while it was negatively correlated with Al traits (Figure 10). The expression and annotation of genes in the above five modules are listed in Table S3-1–S3-5. The module is a highly interconnected set of genes that co-express to function and involve a biological process. However, the genes within a module are not necessarily differentially expressed genes. For instance, 2075 genes were clustered into MEturquoise; none were upregulated, and only a small portion was weakly downregulated by Al and Mn. RRL is the primary indicator for Al and Mn toxicity and tolerance screening. Forty-two genes in MEturquoise were directly related to root development, such as putative auxin transporter-like protein (*LAX3*), which was related to lateral root formation; bifunctional L-3-cyanoalanine synthase (*PALP*), which was related to root hair cell development; germin-like protein (*GLP*), which was related to the regulation of root development; protein root hair defective 3 (*RHD3*); root phototropism protein 3 (*NPH3*); and root-specific lectin (*ChtBD*) (Table S3-5). These genes did not respond to Al and Mn, but provided additive information that was not unfolded by the DEGs.

**Figure 11:**
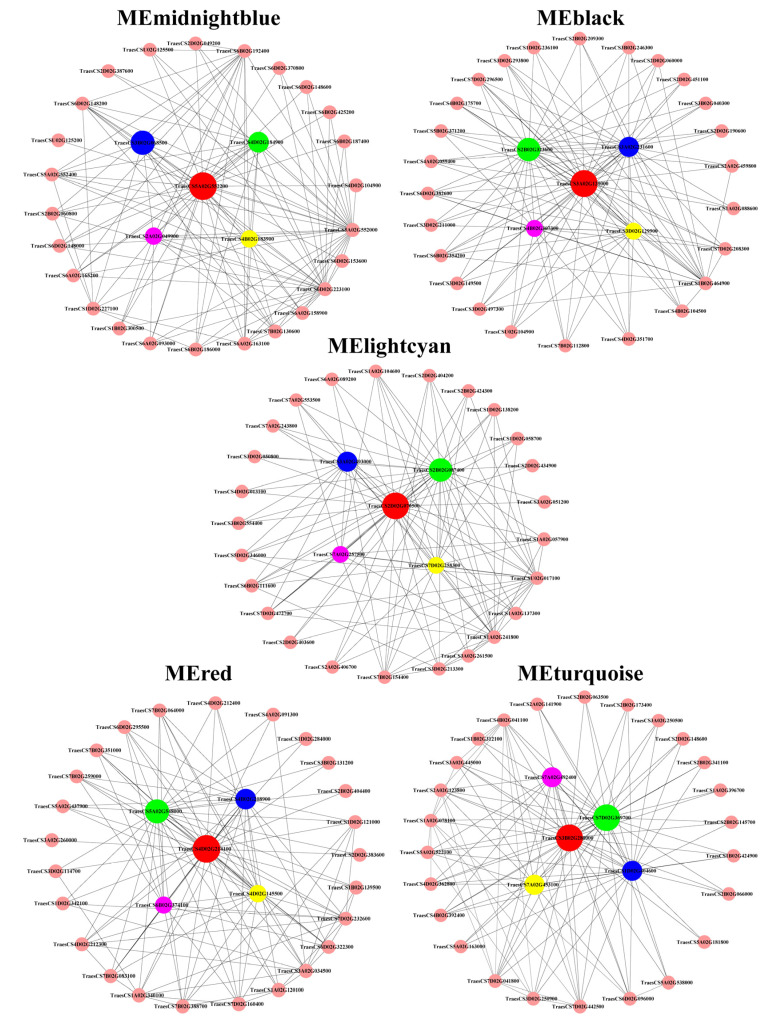
Visualization of gene co-expression network in five modules. Each node represents a gene, and the nodes are connected by lines. The genes at both ends of the lines have similar expression patterns. The top five genes with the highest connectivity were identified as candidate hub genes in each module.

A connectivity analysis was conducted with the top 30 genes with high connectivity in each ME (MEmidnightblue, MEblack, MElightcyan, MEred, and MEturquoise) using Cytoscape to visualize the gene interaction regulation network (Figure 11).

The top five genes with a co-expression weight greater than 0.15, more connectivity, and higher Kme value in the interaction networks were screened. The candidate hub genes in the module were designated based on the annotated gene function and the correlation between the ME and traits. The connectivity, expressions, and annotations of the candidate hub genes are shown in Table 1.

In MEmidnightblue, all of the top five genes encoding nicotianamine synthase were upregulated exclusively by Mn. In MEblack, two genes that functioned as zinc finger transcription factors and three genes that were involved in intracellular trafficking and secretion were upregulated strongly by Al, while they were upregulated marginally by Mn. In MElightcyan, two genes functioned as inorganic ion transporters and were involved in glutathione metabolism; one gene encoding the TCP family transcription factor and one gene that was involved in protein processing in the endoplasmic reticulum presented more connectivities. However, their expressions were constitutive, and not strongly regulated by Al nor Mn. This result provides new insights into the mechanisms underlying Al and Mn toxicity and tolerance beyond differentially expressed genes (DEGs). In MEred, three genes involved in genetic information processing and two genes that encoded HSP20 were significantly upregulated by Al and Mn. In MEturquoise, two genes involved in proton and auxin transport and two genes involved in cell wall biogenesis and phenylpropanoid biosynthesis were slightly downregulated by Al and Mn. These genes may be a hub in their module and play a vital role in regulating the corresponding physiological trait.

## 4. Discussion

### 4.1. Cell Wall Biogenesis and Macromolecule Metabolism Respond Exclusively to Al Stress

The root tip is the first site for aluminum absorption, and aluminum toxicity inhibits the expansion and elongation of root cells and subsequent cell division [2]. Cell walls are the first barrier to Al uptake at the root apex. More than 80% of the total accumulated Al in plant roots is tightly bound to the cell walls, with only a tiny fraction entering the cell sap (cytoplasm), while Mn is mainly distributed in the cell sap (Figure 3). The primary cell wall components, pectin, and hemicellulose can bind Al; the binding of Al to cell walls decreases extensibility and cell elongation and increases rigidity [5]. Al stress significantly increases the contents of cell wall polysaccharides (pectin, hemicellulose I, and hemicellulose II) and the activities of pectin methylesterase and pectin demethylation at the root tips, increasing the fixation of Al by cell walls. The pectin methylation degree is determined by the pectin methylesterase (PME) activity [15]. Xyloglucan is the most active Hemicellulose component in the cell wall; it is biosynthesized in the Golgi apparatus by a series of glycan synthases and glycosyltransferases (XTHs) before being exported to the wall [38]. *XTHs* are considered important factors in controlling the strength and ductility of the cell wall and are extremely sensitive to Al [39]. Becnel et al. (2006) found that at least twenty-one *XTH* members had strong expressions in the root, especially *XTH14*, *XTH15*, and *XTH31* [40]. The root-specific expressions in *XTH5*, *XTH12*, *XTH13*, *XTH14*, *XTH17*, *XTH18*, *XTH19*, *XTH20*, *XTH26*, and *XTH31* were differentially expressed in response to Al [41].

**Figure 12:**
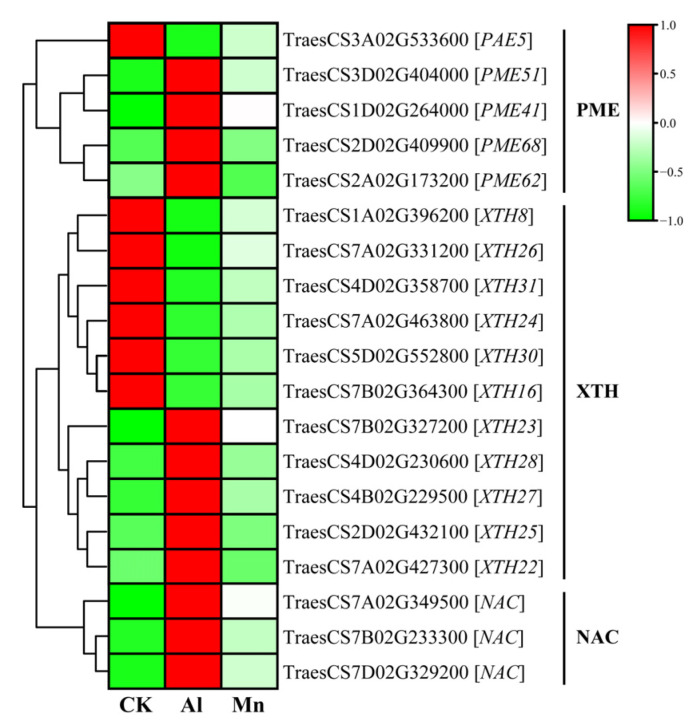
The expression levels of DEGs involved in cell wall biogenesis and macromolecule metabolism under Al and Mn stresses. PAE, PME: Pectin methylesterase; XTH: Glycan synthases and glycosyltransferases; NAC: NAM, ATAF1/2, and cup-shaped cotyledon-2.

This study found that genes regulating cell wall modification were specifically induced by Al stress but not significantly by Mn. Four of the five genes encoding PME were upregulated, while one was downregulated, which may explain the increase in cell wall Al binding (Figure 12). Similarly, *XTHs* were differentially expressed with a divergent pattern: *XTH22*, *XTH23*, *XTH25*, *XTH27*, and *XTH28* were upregulated, while *XTH8*, *XTH16*, *XTH24*, *XTH26*, *XTH30*, and *XTH31* were downregulated. In addition, the NAC transcription factor, which directly binds to the *XTH31* promoter region in *Arabidopsis* [42], was also highly upregulated in our study. In vivo, the localization of XTH activity showed that Al greatly inhibited this enzyme activity within 30 min of exposure, which was concomitant with Al-induced callose deposition in the roots [39]. Under Al stress, plants may increase resistance by reducing the production of xyloglucans through the downregulation of the *XTH* genes, wherein *XTH31* plays a significant role; the expression of other *XTH* genes may be involved in synergistic *XTH31* expression or provide energy [39].

The WGCNA results showed that MEturquoise and MEblack were strongly correlated with Al, while they were weakly correlated with Mn. Numerous genes in the two MEs were involved in synthesizing and transporting cell wall components (Table S3-2 and S3-5). For instance, peroxidase (POD) P7, encoded by the hub gene TraesCS7D02G369700, is mainly involved in clearing root cell ROS and synthesizing lignin. Another hub gene, TraesCS3B02G280000, annotated as V-type proton ATPase bundle B2, regulates the synthesis and transport of lignin monomers (Table 1) [43]. Lignin synthesis is a typical defense mechanism in plant stress response. Lignin accumulates in the cell wall of the root endothelial layer, inhibiting the entry of heavy metals into the xylem or their transport from vascular bundles to the aboveground. *OsSTAR1* and *OsSTAR2*, encoding a nucleotide-binding domain and a transmembrane domain of an ABC transporter, improve Al tolerance in rice through cell wall modification [44,45]. The TraesCS3A02G129000 gene encoding the ABC transporter C family member 3-like was exactly the hub gene in MEblack (Table 1).

The hardening of cell walls has a dual effect: on one hand, it inhibits the elongation of roots, and on the other hand, it prevents harmful metals from entering the cytoplasm. The modification of root cell wall properties can be reversible, so it is worth discussing whether cell wall stiffening is the cause or consequence of root growth inhibition.

### 4.2. Nicotianamine Synthesis Responds Exclusively to Mn Stress

Nicotianamine (NA) is a low-molecular-weight metal-bound ligand that maintains the homeostasis of Fe, Zn, and Mn. It protects cells from metal-induced damage, such as oxidative stress [46]. L-methionine is the precursor for NA biosynthesis, which binds with ATP to synthesize S-adenosylmethionine (SAM). Subsequently, three S-adenosylmethionine molecules are condensed to NA by nicotianamine synthase (NAS). NA is finally converted into 2′-deoxymugineic acids (DMA) and other mugineic acids (MAs) under the catalysis of nicotianamine aminotransferase (NAAT) [47]. The overexpression of *MxNAS2* and *MxNAS3* genes from *M. xiaojinensis* increases the concentration of NA-Mn in the flowers and leaves of tobacco and *Arabidopsis*, respectively [46]. Yellow strip-like protein (YSL) is involved in the uptake, transport, and relocation of metal complexes such as Mn-NA, Mn-HMA, and Mn-DMA to maintain Mn ion balance in plants [6,48].

**Figure 13:**
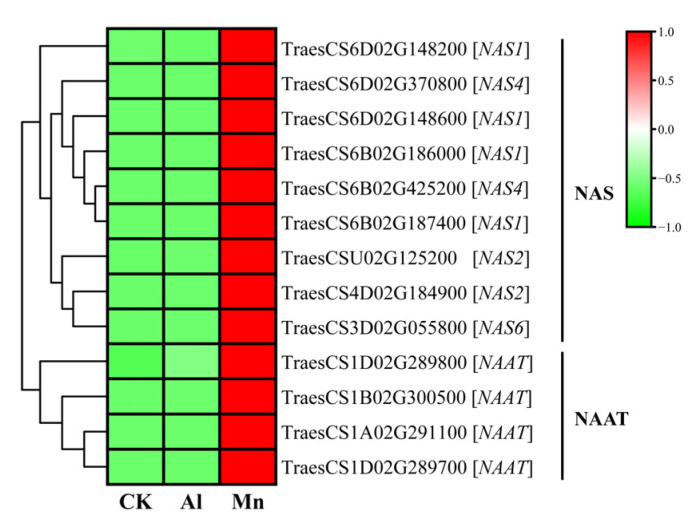
The expression levels of DEGs involved in nicotianamine synthesis under Al and Mn stresses. NAS: nicotianamine synthase; NAAT: nicotianamine aminotransferase.

**Figure 14:**
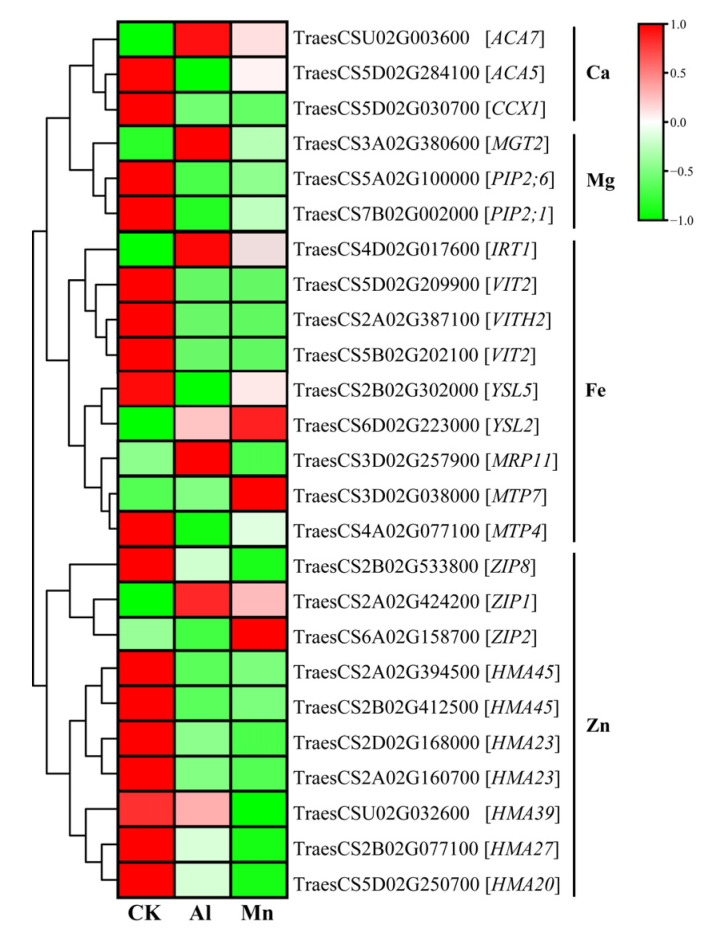
The expression levels of DEGs involved in metal ion transport under Al and Mn stresses. ACA: calmodulin-activated Ca^2+^ pumps; CCX: cation/calcium exchanger; MGT: magnesium transporter; PIP: aquaporin; IRT: iron-regulated transporter; VIT: vacuole iron transporter; YSL: yellow stripe-like protein; MRP: multidrug resistance-associated protein; MTP: metal tolerance protein; ZIP: zinc/iron-regulated transporter protein; HMA: heavy-metal-associated domain.

The results included nine genes encoding NAS, four genes encoding NAAT, and two genes encoding YSL, which were specifically and strongly upregulated by Mn stress but not significantly regulated by Al (Figure 13). For instance, the expressions of *NAS4*, *NAS1*, *NAS2*, and *NAATA* increased by 3040, 420, 342, and 577-fold under Mn stress. In addition, the *YSL2* gene (TraesCS6D02G223000), which transports Mn-NA and Mn-DMA [48,49,50], was also upregulated exclusively by Mn (Figure 14).

WGCNA showed that the genes encoding NAS, NAAT, and YSL2 were all significantly upregulated by Mn in MEmidnightblue; additionally, three genes encoding heavy-metal-associated domain (HMA) and one gene encoding ZIP were identified (Table S3-1). Most importantly, five genes encoding NAS were all included in the top 30 genes with the highest connectivity (Table 1), playing a hub role in the regulatory network to Mn stress.

Increasing the synthesis of NA and its derivatives and enhancing the long-distance transport of Mn-MAS is a specific strategy to tolerate Mn.

### 4.3. Metal Ion Transportation and Accumulation under Al and Mn Stresses

The Al absorption site is located at the root tip, and Al induces a series of transporter genes to coordinate the absorption, transport, and redistribution of Al. NRAMP aluminum transporter 1 (NRAT1) plays a vital role in rice Al tolerance by reducing the level of toxic Al in the root cell wall and transporting Al ions (not Al-citrate complex) into the root cell, where they are ultimately sequestered in the vacuole [26,51]. The ALS protein family is responsible for transporting excess Al to the vacuole for isolation and thus enhancing tolerance to Al in rice [52], tea [53], and buckwheat [54]. In this study, under Al stress, two nodulin 26-like intrinsic protein (NIP) genes, *TaNIP1;1* (TraesCS7B02G122600) and *TaNIP1;2* (TraesCS7A02G215700), were significantly upregulated by Al stress. This may promote the transport of the malate–Al complex (not Al ion) from the cell wall to the symplasm and further transport to the leaf vacuole for segregation [14,45,55]. WGCNA showed that MEblack and MElightcyan were significantly correlated with the content and distribution of Al. Some genes upregulated in MEblack may be involved in Al absorption, including those annotated as ABC transporters and one gene encoding NRAMP (Table S3-2).

Manganese is essential for plant growth, with a narrow concentration window between deficiency and toxicity. Therefore, Mn transport systems should be tightly regulated in plants. Plants must deliberately control the uptake, transportation, and distribution of manganese to prevent toxicity caused by excessive amounts of this element. Transporters play a vital role in these processes and thus help plants to tolerate Mn toxicity. Mn absorption occurs in the mature root zone and can be easily transported through the xylem to the shoot [56]. The *Arabidopsis* root plasma membrane-localized transporter natural resistance-associated macrophage protein (AtNRAMP1) mediates Mn^2+^ absorption. Two members of the zinc/iron-regulated transporter protein (ZIP) family, AtZIP1 and AtZIP2, are involved in the transport of Mn from the root to stem [8,57]; OsYSL2 is responsible for the long-distance transport and distribution of Mn [50]. In this study, the genes of *TaYSL2* (TraesCS6D02G223000), *TaZIP1* (TraesCS2A02G424200), and *TaZIP2* (TraesCS6A02G158700) were significantly upregulated under Mn stress, and thus increased Mn absorption (Figure 14). Mn shares some ion channel proteins with other metal ions. For instance, OsNRAMP5, a plasma membrane protein, is involved in constitutive Fe and Mn uptake and inducive transport during flowering and seed development [58]. In the present study, the expression of reported multifunctional transporter genes, such as *CAX2*, *CCX3*, *MTP9*, *MTP10*, *MTP11*, *MTP11*, *NRAMP3*, *NRAMP5*, and *YSL6* [8], were not regulated by Mn. The diversity of genes involved in Mn uptake and transport and the versatility of genes lead to the complexity of the regulation of Mn ion balance.

Al and Mn inhibit root growth, subsequently leading to the deprivation of essential nutrient elements. Due to its higher charge, the Al ion inhibits the absorption of bivalent cations (Ca^2+^, Mg^2+^, Fe^2+^, and Zn^2+^) more strongly than other toxic ions [1]. It removes bivalent cations from the plasma membrane and cell wall [59]. High concentrations of Mn^2+^ compete with other nutrient cations, thus hindering their uptake [8]. On the other hand, roots can improve tolerance to Al and Mn toxicity stresses by controlling the uptake of mineral nutrients and maintaining ion homeostasis, including calcium, magnesium, zinc, and iron [6,8,10].

Our results showed that Al and Mn stresses decreased the Ca, Mg, and Zn contents, and decreased the absorption and accumulation of Fe by Al, while Mn exerted the opposite effects. Al and Mn significantly downregulated one gene (TraesCS5D02G030700) encoding the cation/calcium exchanger (CCX1), contributing to the decrease in the Ca content. Plants utilize calmodulin-activated Ca^2+^ pumps (ACA) at the plasma membrane, endoplasmic reticulum, and vacuole [60]. The *ACA7* (TraesCSU02G003600) and *ACA5* (TraesCS5D02G284100) might play essential roles in altering the production of cellular Ca^2+^ signals and thus mediate adaptive responses (Figure 14).

Al and Mn shared hydrating ionic radii with Mg, competing for binding sites on apoplasm and transporters in the plasma membrane [61]. Aquaporin (PIP) regulates water and Mg^2+^ transport [62]. Two *PIPs* (TraesCS5A02G100000 and TraesCS7B02G002000) were significantly downregulated, which was related to a decrease in Mg^2+^ absorption. The decrease in the Mg content under Al and Mn stress may be the reason for the decrease in the chlorophyll content (SPAD). The overexpression of *MGT1* can enhance Al tolerance [63]. The expression of one *MGT* gene (TraesCS3A02G380600) was upregulated under Al stress but not Mn due to an adaptive response related to the regulation of membrane potential balance by H^+^-ATPase activity under Al stress (Figure 14).

Fe is an essential trace element for plant growth and development. However, free Fe in cells produces toxic ROS through the Fenton reaction [64], necessitating strict control of iron homeostasis through various strategies. Al stress increased Fe absorption in tea plants by upregulating Fe ion transporter genes, including *FRO*, *YSL*, and *IRT*, maintaining Fe homeostasis [53]. Mn and Fe have antagonistic solid effects, and excessive Mn^2+^ takes advantage of Fe on the binding of Fe/Mn co-transporters [8,65]. Most of the CDF/MTP and VIT families can transport Fe^2+^ and Mn^2+^, and excess Mn^2+^ blocks the absorption of Fe^2+^ [8]. In this study, three *VIT* family genes and one *YSL5* gene were significantly downregulated under Al and Mn stresses, reducing Fe absorption. One *IRT1* gene (TraesCS4D02G017600) and one *MGP* gene (TraesCS3D02G257900) were upregulated by Al stress but downregulated by Mn stress (Figure 14). These results indicate that Al stress increased Fe^2+^ absorption by upregulating *IRT* and *MRP*. *MTP4* (TraesCS4A02G077100) and *MTP7* (TraesCS3D02G038000) were downregulated and upregulated by Mn stress, respectively, which affected the absorption and transport of Mn and Fe.

High active Al^3+^ competes with Zn^2+^ for binding sites on the root plasma membrane, and excess Mn^2+^ shares a similar transporter with Zn^2+^, resulting in the plant inhibition of zinc absorption [1,6]. Heavy-metal-associated domain (HMA) and ZIP proteins are involved in the absorption and transport of Zn [8]. Our study indicated that seven genes from the *HMA* family were significantly downregulated under Al and Mn stresses. The expression of genes from the *ZIP* family was diverse, with the significant upregulation of *ZIP1* (TraesCS2A02G424200) and the downregulation of *ZIP8* (TraesCS2B02G533800) under Al and Mn stresses. In addition, *ZIP2* (TraesCS6A02G158700) was upregulated by Mn stress but downregulated by Al, which is favorable for the absorption of Mn and Zn (Figure 14).

In MEturquoise (Table S3-5), the genes encoding the calcium load-activated calcium channel (CAEEL); the Fe transporters, VIT and YSL; and the Zn transporter, HMA, were downregulated by Al and Mn. In contrast, Zn transporter genes (ZIP) in the MEred were upregulated (Table S3-4). MEturquoise was positively correlated with RRL, while MEred was negatively correlated with RRL. Reducing the relative root length may decrease the absorption area and gene expression levels, ultimately inhibiting nutrient uptake.

### 4.4. Phenylpropanoid Biosynthesis under Al and Mn Stresses

**Figure 15:**
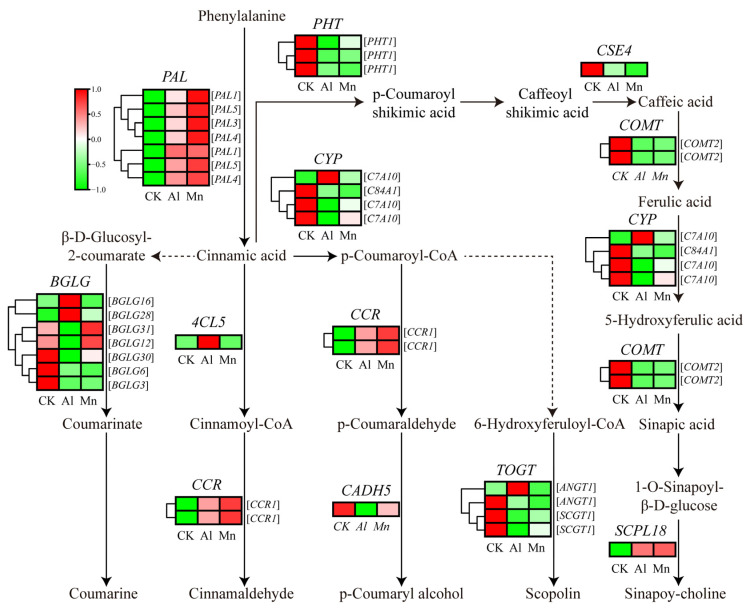
The expression levels of DEGs involved in phenylpropanoid metabolism under Al and Mn stresses. The gene expression values of different samples or experimental conditions were normalized and scale-mapped to [−1,1] from minimum (green) to maximum (red). PAL: Phenylalanine ammonia-lyase; 4CL: 4-coumarate--CoA ligase; CCR: Cinnamoyl-CoA reductase; BGLG: Beta-glucosidase; CYP: Cytochrome P450; CAD: Cinnamyl alcohol dehydrogenase; TOGT: Anthocyanin 3′-O-beta-glucosyltransferase; PHT: Putrescine hydroxycinnamoyl transferase; CSE: Caffeoylshikimate esterase; COMT: Tricetin 3′,4′,5′-O-trimethyltransferase; SCPL: Serine carboxypeptidase-like protein.

The KEGG results indicated that genes involved in phenylalanine metabolism commonly responded to Al and Mn, and most genes involved in phenylpropanoid metabolism were downregulated by both Al and Mn; some genes involved in flavonoid metabolism specifically responded to Al (Figure 8). There is the same expression trend of genes involved in the metabolism pathway from phenylalanine to sinapoy-choline and scopolin under Al and Mn, with the downregulation of seven genes encoding phenylalanine ammonia-lyase (PAL), three genes encoding putrescine hydroxycinnamoyltransferase (PHT), one gene encoding caffeoylshikimate esterase (CSE), two genes encoding tricetin 3′,4′,5′-O-trimethyltransferase (COMT), three genes encoding Cytochrome P450 (CYP), and three genes encoding Anthocyanin 3′-O-beta-glucosyltransferase (TOGT), and the upregulation of one gene (TraesCS3A02G329200) encoding serine carboxypeptidase-like (SCPL); however, one gene (TraesCS7A02G398900) encoding CYP45073A and one gene (TraesCS5B02G522400) encoding TOGT1 were exclusively upregulated by Al. Two genes (TraesCS6D02G136600 and TraesCS6A02G147300) encoding cinnamoyl-CoA reductase (CCR) were upregulated by Al and Mn. Among seven genes encoding beta-glucosidase (BGLG), three were downregulated by Al and Mn, *BGLG31* (TraesCS5D02G302700) and *BGLG6* (TraesCS4A02G056400) showed significant upregulation by Al, and *BGLB12* (TraesCS2A02G329000) and *BGLB3* (TraesCS5D02G404700) were upregulated by Mn but downregulated by Al. One gene (TraesCS3D02G388100) encoding 4-coumarate-CoA ligase (4CL) and one gene (TraesCS7D02G328300) encoding cinnamyl alcohol dehydrogenase (CAD) were exclusively up- and downregulated by Al, respectively (Figure 15).

Phenylpropanoid biosynthesis is an important pathway that produces plant secondary metabolites, such as phenylpropanoids, flavonoids, and diterpenoids, which are secreted in response to metal ions [66,67]. PAL catalyzes phenylalanine cinnamic acid, the first rate-limiting enzyme in the phenylalanine biosynthesis pathway [68]. Our results showed that genes involved in the pathway from cinnamic acid to sinapoy-choline and scopoline were downregulated, while *PAL* and *CCR* were upregulated by both Al and Mn. Similar to our study, genes encoding PAL and CCR were upregulated in phenylpropanoid metabolism and are activated to counteract Mn stress in *Arabis paniculate* [69]. In *Populus euphratica*, the content of phenylalanine and the antioxidant enzyme activity increases under Mn stress [70]. Phenylpropanoid metabolism exhibits different expression patterns in the roots and leaves under Mn stress in *Stylosanthes*. The expressions of *PAL1* and chalcone-flavanone isomerase (*CFI*) were upregulated in the leaves, while *PAL2* and chalcone synthase (*CHS*) were downregulated in the roots [71]. It was also found that phenylpropanoid biosynthesis and phenylalanine metabolism played a critical role in the defense against aluminum [72]. Lettuce protected roots from oxidative damage by upregulating phenylalanine ammonia-lyase (PAL), cinnamate 4-hydroxylase, and 4-coumarate coenzyme A ligase [73]. These results suggest that phenylpropanoid biosynthesis enhanced the antioxidant capacity for the tolerance against both Al and Mn.

In addition, Al specifically upregulated *4CL* and *CYP*, which are involved in the synthetic precursors of flavonoid metabolism, p-coumaroyl-CoA, and cinnamoyl-CoA. The KEGG results showed that Al exclusively downregulated some genes involved in flavonoid metabolism, and two genes (TraesCS2D02G582000 and TraesCS4A02G003100) encoding tryptamine benzoyltransferase 1 (TBT1) and one gene (TraesCS2A02G482200) encoding anthocyanidin reductase (ANR) were exclusively upregulated (Table S2-3). The flavonoid biosynthesis site is located at the upper end of the surface cells in the plants’ root growth area and participates in internal and external Al detoxification by forming solid complexes with toxic Al ions [74]. Alfalfa increased the resistance to Al stress by increasing flavonoid accumulation in roots and secretion from the root tips [75]. Our results suggest that wheat has a tolerance to Al by promoting flavonoids to chelate Al ions.

### 4.5. Signaling in Roots under Al and Mn Stresses

According to the KEGG results, the majority of genes involved in signaling, including those related to the MAPK signaling pathway, plant–pathogen interaction, and plant hormones like IAA, ETH, and JA, exhibited similar expression patterns. However, the specificity of ZT and BR in response to Al and Mn was noticeable.

**Figure 16:**
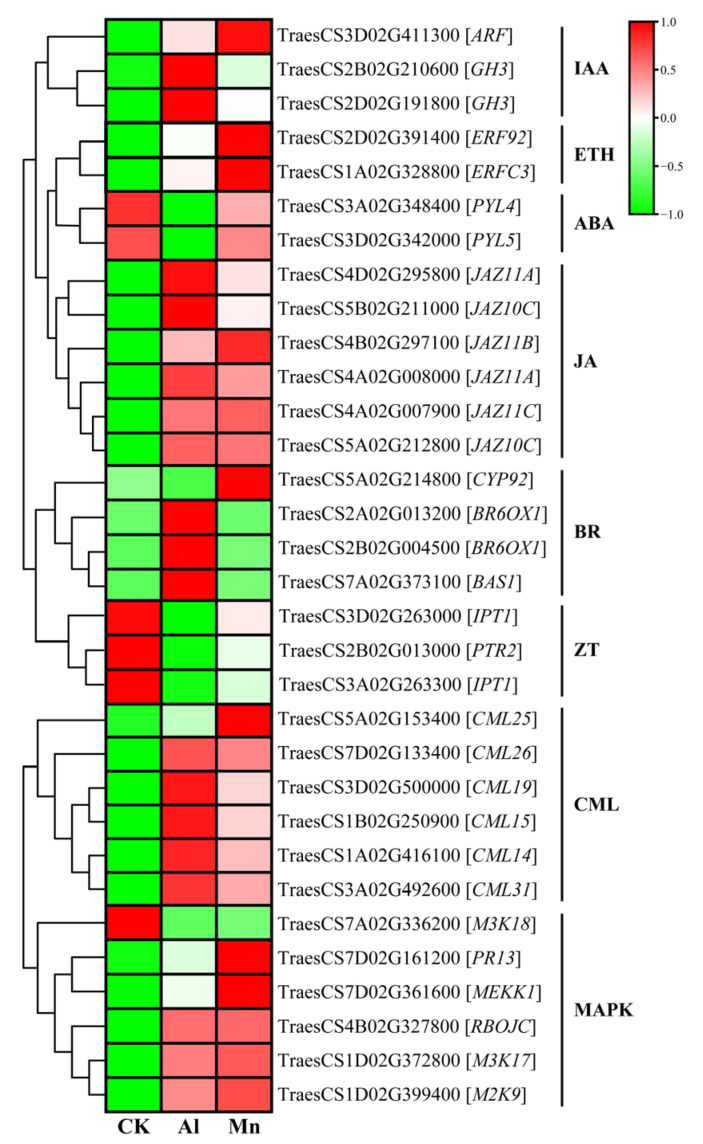
The expression levels of DEGs involved in signaling under Al and Mn stresses. ARF: auxin-responsive factor; GH: indole-3-acetic acid-amido synthetase GH; ERF: ethylene-responsive transcription factor; PYL: abscisic acid receptor PYL; JAZ: jasmonate ZIM domain protein; BR: brassinosteroid; IPT: adenylate isopentenyltransferase; PTR: protein NRT1/PTR family; CML: calmodulin-like protein; M3K, M2K, MEKK: mitogen-activated protein kinase; PR: pathogenesis-related protein; RBOH: respiratory burst oxidase homolog protein.

Genes that are commonly expressed in plant hormones (Figure 8), including two genes (TraesCS2B02G210600 and TraesCS2D02G191800) encoding indole-3-acetic acid-amido synthetase GH3, one gene (TraesCS3D02G411300) encoding auxin-responsive factor (ARF), two genes (TraesCS2D02G391400 and TraesCS1A02G328800) encoding ethylene-responsive transcription factor (ERF), and six genes encoding jasmonate ZIM domain protein (JAZ), were upregulated. Two genes (TraesCS3A02G348400 and TraesCS3D02G342000) encoding abscisic acid receptor PYL were downregulated by both Al and Mn, with a pronounced downregulation by Al than Mn. While some genes exclusively responded to Al or Mn, three genes involved in brassinosteroid (BR) were exclusively upregulated by Al, while one gene (TraesCS5A02G214800) encoding CYP92A6 was exclusively upregulated by Mn. Furthermore, three genes involved in zeatin (ZT) were exclusively downregulated by Al, including two genes (TraesCS3A02G263300 and TraesCS3D02G263000) encoding adenylate isopentenyl transferase 1 (IPT) and one gene (TraesCS2B02G013000) encoding protein NRT1/ PTR family 8.5 (PTR) (Figure 16).

IAA and JA affected the antioxidant defense mechanisms. They altered the cellular redox homeostasis for Al and Mn, showing the commonality of plant hormones in response to Al and Mn stresses [76]. In addition, plants respond to both stresses in some specific mechanism. The primary lesion of Al toxicity is the rapid inhibition of root elongation, with plant hormones primarily reacting to Al stress in the roots [10]. Genes involved in BR and ZT were specifically reposed to Al, which might have contributed to root growth and organic acid. The activated ethylene signal by Al promoted IAA or cytokinin (CTK) accumulation and led to a modification in the cell wall structure through auxin-responsive factors (ARFs). It was reported that BZ signaling is involved in the expressions of *ALMT1* and *STOP*. It also stimulated the synthesis of the jasmonic acid signaling to modulate microtubule (MT) polymerization to regulate root growth [15]. Auxin-regulated GmMATE and H^+^-ATPase enhance Al tolerance by modifying the cell walls and promoting citrate exudation. Abscisic acid (ABA) was found to mediate the Al-induced citrate exudation in soybean and ethylene-mediated JA signal-regulated ALMT1-controlled malate exudation [15]. These results suggest that plants participating in Al tolerance mainly focus on the roots. While the changes in plant hormone content were influenced by Mn accumulation, moderate Mn increased the IAA, ZT, and 6-BA (6-benzylaminopurine) contents but decreased the ABA content in the leaves. A high Mn accumulation significantly increased the leaf JA and ABA contents, which inhibited plant growth and stimulated stress tolerance [77]. Mn enhanced the resistance to Mn toxicity based on IAA and ABA synthesis by regulating growth and scavenging ROS for antioxidants and Mn transporter [78].

Most genes involved in the MAPK signaling pathway and plant–pathogen interaction expressed the same trend under Al and Mn stresses. Six genes encoding calmodulin-like proteins (CML) and one gene (TraesCS4B02G327800) encoding respiratory burst oxidase homolog protein (RBOH) were significantly upregulated by Al and Mn. Among three genes encoding mitogen-activated protein kinase (MAPK), *M2K9* and *M3K17* showed significant upregulation, while Al and Mn stresses downregulated *M3K18*. Moreover, one gene (TraesCS7D02G161200) encoding a pathogenesis-related protein (PRP) and *MEKK1* was exclusively upregulated by Mn (Figure 16).

Signaling, such as Ca^2+^, ROS, and NO, is a late response to Al stress. Al promoted the binding of Ca^2+^ to CML24, leading to malate exudation by regulating the expressions of *STOP1*, *WRYK46*, and *ALMT1* [79]. The MAPK pathway positively regulates *STOP1*, thus conferring Al resistance [80]. Similarly, the response of these signals is vital for Mn. Mn^2+^ increased the catalase activity by regulating mitogen-activated protein kinases (MAPKs) and calmodulins in the presence of calcium to reduce Mn toxicity [81]. The production of one of the ROS, hydrogen peroxide (H_2_O_2_), is often generated contemporaneously with NO, and is regulated by *CML* and *RBOH*, respectively. NO was shown to play a familiar role in reducing Al toxicity by regulating the root growth and antioxidant capacity in wheat roots [82,83] and alleviating Mn toxicity by preventing oxidative stress in rice [84].

### 4.6. Transcription Factors Regulate Wheat Tolerance to Al and Mn Stresses

The GO annotation analysis showed that transcription factors (TFs) were predominantly upregulated by Al and Mn (Figure 7). Transcription factors are the terminal points of stress signal transduction and molecular switches for downstream gene expression [85]. Numerous TFs, such as WRKY, MYB, GATA, bZIP, bHLH, and ERF, have been reported to regulate Al and Mn stress responses [86,87,88]. For instance, sensitive to proton toxicity (STOP1) and its rice homolog Al resistance transcription factor (ART1) are both members of the C2H2 zinc finger family, which enhances Al tolerance by regulating *ALMT1* [89,90]. The overexpression of *GsERF1* enhances aluminum tolerance in *Arabidopsis thaliana* through ethylene-mediated pathways and ABA signaling pathways [91]. Similarly, ZmbHLH105 improves tolerance to manganese stress by regulating antioxidant mechanism-mediated ROS clearance and Mn/Fe-related transporter expression in plants [92]. Some transcription factors that regulate genes associated with aluminum (Al) tolerance may also have similar or opposite roles in manganese (Mn) tolerance. For instance, C2H2 transcription factors activate Al-tolerant genes, including glutamate dehydrogenase 1 (*GDH1*), pectin methylesterase inhibitor (*PMI*), malic enzyme (*ME*), aluminum-activated malate transporter (*ALMT*), multidrug and toxic compound exclusion protein (*MATE*), and tonoplast dicarboxylate transporter (*TDT*) [93,94]. These homologous genes were upregulated by Mn toxicity [95]. WRKY46 is a negative regulator of *ALMT1* in *Arabidopsis thaliana*, and the destruction of WRKY46 leads to increased malate secretion, resulting in Al tolerance [96]. However, *WRKY* gene transcripts in stylo are enhanced by Mn stress [95], suggesting that there are conserved and divergent regulatory networks mediated by WRKY-mediated gene expression under Al and Mn toxicity.

An expression analysis showed that 640 TFs were significantly regulated by at least one stress, covering 29 TFs families (Table 2). The MYB family accounted for the most, followed by the WRKY, AP2, bHLH, and NAC families. The differentially expressed TFs from the BES1, GRF, HD-ZIP, RAV, TCP, and NF-YA families were exclusively regulated by Al. In contrast, two TFs from calmodulin-binding transcription activators (CAMTAs) and ethylene-insensitive 3-like (EIL) families were exclusively regulated by Mn, indicating the specificity of these TFs in sensing stress and regulating downstream genes. A total of 455 differentially expressed TFs were found under Al stress, comprising 291 upregulated and 82 downregulated TFs. Similarly, 373 TFs were differentially expressed under Mn stress, including 414 upregulated and 212 downregulated TFs. There were far more upregulated TFs than downregulated TFs by either Al or Mn, suggesting that wheat tends to activate TFs to adapt to toxicity. Numerous differentially expressed TFs were found under only one stress, with 267 TFs exclusively regulated by Al and 185 by Mn. A total of 188 differentially expressed TFs, including 156 upregulated and 32 downregulated TFs, were identified under two stresses in the same trend, demonstrating that the most co-expressed TFs function similarly under Al and Mn stresses.

## 5. Conclusions

The current study analyzed the physiology and transcriptomics of roots from wheat seedlings that were tolerant to Al and Mn stresses. The results showed that Al was mainly fixed in the root cell walls, regulated by *PEMs* and *XTHs*. Mn was distributed primarily on the cell saps and then transferred to the shoots by NA and YSL. The expression of metal cation transporter genes was altered by Al and Mn, leading to a reduced absorption of Ca, Mg, and Zn. Additionally, Mn decreased Fe absorption, while Al had the opposite effect. The identified DEGs associated with commonality and specific pathways based on the KEGG enrichment analysis might represent an adaptive strategy of wheat to cope with Al and Mn. Numerous genes related to TFs, such as MYB, WRKY, and AP2 families, were activated to tolerate Al and Mn stresses. In addition, WGCNA identified the hub genes *PODP7*, *VATB2*, and *ABCC3* for Al tolerance and *NAS* for Mn tolerance. For these reasons, a further study is recommended to determine the ability of pyramid genes to breed multi-tolerant varieties, to understand the interaction of TFs with other proteins, and to regulate the target genes.

## Figures and Tables

**Table 1:** Connectivity, expressions, and annotations of candidate hub genes’ correlation between ME and traits.

Module	Gene_id	Connectivity	Kme Value	Fold Change		Gene Description
				Al/CK	Mn/CK	
midnightblue	TraesCS5A02G552000	50.146	0.986	0.892	124.101	Nicotianamine synthase
	TraesCS3B02G068500	50.064	0.986	0.830	329.475	Nicotianamine synthase
	TraesCS4D02G184900	50.032	0.994	0.694	53.203	Nicotianamine synthase
	TraesCS4B02G183900	49.729	0.987	0.429	178.986	Nicotianamine synthase
	TraesCS2A02G049900	49.645	0.985	0.486	343.721	Nicotianamine synthase
black	TraesCS3A02G129000	109.335	0.975	2.330	1.080	ABC transporter C family member 3-like
	TraesCS2B02G323600	105.743	0.969	3.924	1.598	RING-type E3 ubiquitin transferase; zinc finger of C3HC4-type
	TraesCS3A02G231600	105.472	0.970	6.459	1.459	Homeobox-leucine zipper protein HOX3
	TraesCS3D02G129900	104.621	0.966	2.711	1.007	ABC subfamily C transporter
	TraesCS4B02G307300	104.457	0.969	7.454	1.341	Intracellular trafficking, secretion, and vesicular transport
lightcyan	TraesCS2D02G070500	33.891	0.967	1.597	0.592	Inorganic ion transport; ascorbate peroxidase; glutathione metabolism
	TraesCS2B02G087400	32.281	0.955	1.508	0.604	Inorganic ion transport; ascorbate peroxidase; glutathione metabolism
	TraesCS3A02G493000	30.610	0.944	1.497	0.660	Function unknown hypothetical protein TRIUR3
	TraesCS7D02G258300	29.417	0.943	1.921	0.560	Protein processing in endoplasmic reticulum; hydrolase
	TraesCS7A02G257500	29.376	0.941	1.797	0.594	TCP family transcription factor; hydrolase
red	TraesCS4D02G214100	226.124	0.982	5.440	4.174	Positive regulation of mRNA splicing via spliceosome
	TraesCS5A02G548000	225.290	0.980	40.422	35.575	BAG family molecular chaperone regulator 6; IQ calmodulin-binding motif
	TraesCS4B02G208900	225.095	0.975	14.932	12.665	Nucleotide exchange factor Fes1B
	TraesCS4D02G145500	223.512	0.965	403.199	310.521	Hsp20/alpha crystallin family
	TraesCS6B02G374100	222.531	0.970	81.004	57.046	Hsp20/alpha crystallin family
turquoise	TraesCS7D02G369700	748.196	0.989	0.301	0.412	Peroxidase P7; phenylpropanoid biosynthesis
	TraesCS3B02G280000	728.892	0.985	0.434	0.626	Proton-transporting V-type ATPase
	TraesCS1D02G404600	727.511	0.984	0.346	0.525	Plant-type secondary cell wall biogenesis; Fasciclin-like arabinogalactan protein
	TraesCS7A02G453100	727.024	0.980	0.260	0.448	Function unknown
	TraesCS7A02G492400	722.960	0.986	0.307	0.517	Auxin efflux carrier component; intracellular trafficking, secretion, and vesicular transport

**Table 2:** Statistics of differentially expressed transcription factors under Al and Mn stresses.

TFs Family	Al_up		Al_down		Al_nr		Total
	Mn_up	Mn_nr	Mn_down	Mn_nr	Mn_up	Mn_down	
MYB	30	30	9	24	14	13	120
WRKY	35	21	0	3	54	1	114
AP2	16	34	0	12	20	3	85
bHLH	7	6	11	8	21	4	57
NAC	8	19	4	3	4	7	45
HSF	25	5	0	2	2	0	34
bZIP	0	17	5	1	1	9	33
HB	2	16	1	2	0	3	24
GRAS	5	7	0	3	8	0	23
B3	3	6	0	6	1	1	17
LBD	3	3	0	3	3	5	17
Dof	2	4	0	2	2	1	11
MIKC	3	4	0	0	2	1	10
C2H2	4	3	0	2	0	0	9
SRS	6	3	0	0	0	0	9
DBB	2	0	2	1	0	1	6
GATA	0	2	0	2	0	1	5
M_type	0	2	0	1	1	0	4
ARF	1	3	0	0	0	0	4
ZF-HD	3	0	0	0	0	0	3
GRF	0	0	0	2	0	0	2
BES1	0	1	0	0	0	0	1
HD-ZIP	0	1	0	0	0	0	1
RAV	0	1	0	0	0	0	1
EIL	0	0	0	0	1	0	1
TCP	0	1	0	0	0	0	1
CAMTA	0	0	0	0	1	0	1
FAR1	1	0	0	0	0	0	1
NF-YA	0	1	0	0	0	0	1
Total	156	190	32	77	135	50	640

Notes: Al (Mn)_up: upregulated by Al (Mn); Al (Mn)_down: downregulated by Al (Mn); Al (Mn)_ns: did not respond to Al (Mn).

## Data Availability

The original contributions presented in this study are available in the article or Supplementary Materials. The RNA-seq raw data can be found on the NCBI repository, accession number PRJNA1031207.

## Supplementary Materials

The following supporting information can be downloaded at https://www.mdpi.com/article/10.3390/cimb46010024/s1, Figure S1: Network topology of different soft threshold powers; Table S1: Primers used for qRT-PCR; Table S2: Expression and functional annotation of DEGs in response to Al and Mn (6 sheets contained); Table S3: Expression and functional annotation of genes in 5 MEs (5 sheets contained).
