# Supplementary figures and images for: Physiological and Transcriptomic Analyses Reveal Commonalities and Specificities in Wheat in Response to Aluminum and Manganese
Source: Curr Issues Mol Biol. 2024 Jan 2;46(1):367–97. doi: 10.3390/cimb46010024 (PMC10814679; doi:10.3390/cimb46010024)

Scale indepence

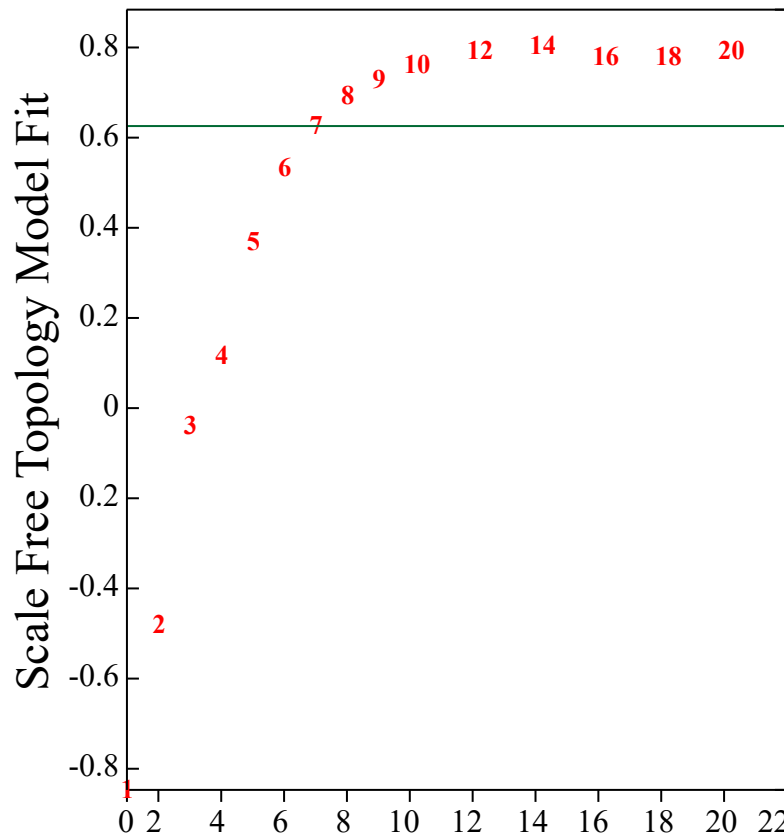

Mean Connectivity

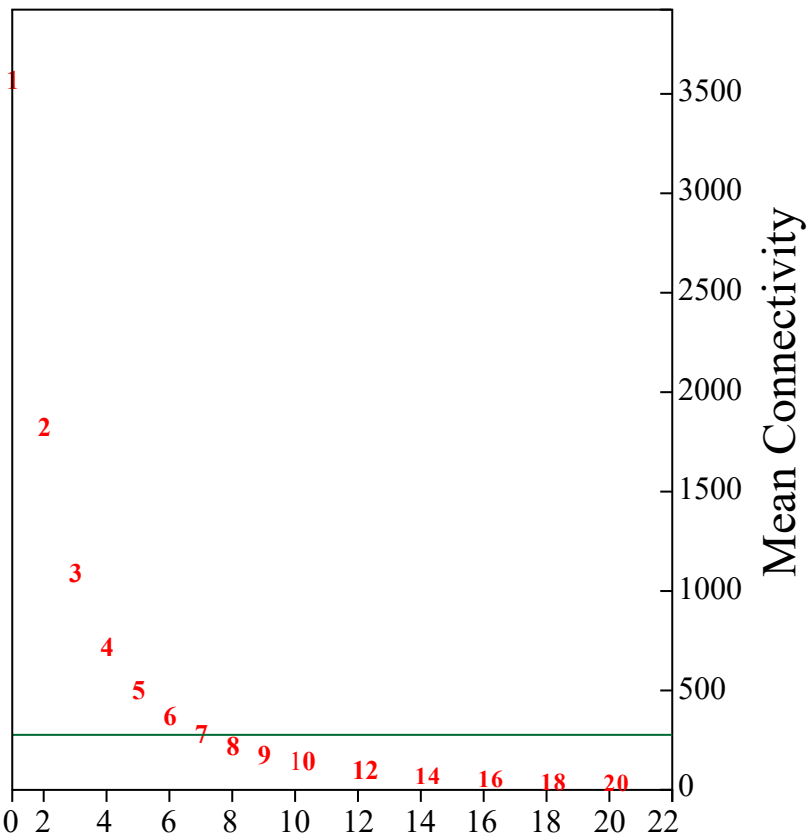

Soft Threshold (power)

Supplement: Supplementary file 1 [file cimb-46-00024-s001.zip › FigureS1-Network topology of different soft threshold powers.pdf]
